# Pharmaceutical Approaches on Antimicrobial Resistance: Prospects and Challenges

**DOI:** 10.3390/antibiotics10080981

**Published:** 2021-08-14

**Authors:** Firzan Nainu, Andi Dian Permana, Nana Juniarti Natsir Djide, Qonita Kurnia Anjani, Rifka Nurul Utami, Nur Rahma Rumata, Jianye Zhang, Talha Bin Emran, Jesus Simal-Gandara

**Affiliations:** 1 Faculty of Pharmacy, Hasanuddin University, Makassar 90245, Sulawesi Selatan, Indonesia; andi.dian.permana@farmasi.unhas.ac.id (A.D.P.); nanajuniarti@unhas.ac.id (N.J.N.D.); qanjani01@qub.ac.uk (Q.K.A.); rifkanurulutami@unhas.ac.id (R.N.U.); n.rumata@yahoo.com (N.R.R.); 2 Medical Biology Centre, School of Pharmacy, Queen’s University Belfast, Belfast BT9 7BL, UK; 3 Institute of Pharmaceutical Science, King’s College of London, London SE1 9NH, UK; 4 Sekolah Tinggi Ilmu Farmasi Makassar, Makassar 90242, Sulawesi Selatan, Indonesia; 5 Key Laboratory of Molecular Target & Clinical Pharmacology and the State & NMPA Key Laboratory of Respiratory Disease, School of Pharmaceutical Sciences & The Fifth Affiliated Hospital, Guangzhou Medical University, Guangzhou 511436, China; jianyez@163.com; 6 Department of Pharmacy, BGC Trust University Bangladesh, Chittagong 4381, Bangladesh; 7 Nutrition and Bromatology Group, Department of Analytical and Food Chemistry, Faculty of Food Science and Technology, University of Vigo–Ourense Campus, E32004 Ourense, Spain

**Keywords:** antimicrobial resistance, antimicrobials, pharmaceutical preparations, drug delivery

## Abstract

The rapid increase in pathogenic microorganisms with antimicrobial resistant profiles has become a significant public health problem globally. The management of this issue using conventional antimicrobial preparations frequently results in an increase in pathogen resistance and a shortage of effective antimicrobials for future use against the same pathogens. In this review, we discuss the emergence of AMR and argue for the importance of addressing this issue by discovering novel synthetic or naturally occurring antibacterial compounds and providing insights into the application of various drug delivery approaches, delivered through numerous routes, in comparison with conventional delivery systems. In addition, we discuss the effectiveness of these delivery systems in different types of infectious diseases associated with antimicrobial resistance. Finally, future considerations in the development of highly effective antimicrobial delivery systems to combat antimicrobial resistance are presented.

## 1. Introduction

The rise of antimicrobial resistance (AMR) has become a significant threat to the global community over recent decades. Generally, AMR is defined as a condition in which microorganisms (bacteria, fungi, viruses, and parasites) can live and grow in the presence of antimicrobial agents that were previously reported to be effective against these microorganisms. In more specific terms, there are several patterns of AMR that have been documented: multidrug-resistant (MDR), extensively drug-resistant (XDR), and pan drug-resistant (PDR). MDR is defined as the acquired resistance of microbe(s) to at least one agent in three or more antimicrobial groups and XDR is defined as microbial resistance to at least one agent in all but two or fewer antimicrobial categories. Lastly, once microbial resistance is developed to agents in all antimicrobial groups, the term PDR is used [1].

MDR-bacteria kill around 700,000 people globally in a year, while 230,000 deaths per year are caused by MDR tuberculosis [2]. In its 2020 Global Report on Tuberculosis, the World Health Organization (WHO) reported that approximately 10.0 million people contracted TB in 2019, and most cases were recorded in South East Asia, accounting for 44% of total global cases. In 2019, about 12,000 XDR-TB cases were reported [3], around 1.5 times higher than XDR-TB cases in 2015, which accounted for approximately 8000 cases [4]. PDR cases in Gram-negative bacteria were reported to show a high mortality rate (21–70%) out of a total of 81 reported cases; around 47 data were documented from the last five years [5]. However, it was suggested that PDR infection can still be controlled at this time [5].

In addition to the existing resistance cases, emerging AMR in microorganisms previously not a concern is also a severe problem. MDR-*Candida auris* was once not included in the threat level in the 2013 Centers for Disease Control and Prevention (CDC) report. Six years later, this fungus was reported as one of the urgent MDR microbials in the 2019 CDC report. *Candida auris* will be the severe next-level threat for AMR in the clinic since it remains unnoticed in microbiology laboratories and is often misidentified by conventional biochemical diagnostic tests [6]. This pathogen has a high resistance rate towards antifungals used in clinics—including azoles, echinocandins, and polyenes—making this fungus a potential pan-resistant strain [7]. The exact same change in threat level status also occurred in Acinetobacter, which was previously classified as urgent but became severe in the 2019 CDC report [8]. Such changes in the AMR category are driven by the ease of spread, the danger posed by the microorganism, the lack of available antibiotics, and the development of antibiotics to deal with this infection [8].

The declining availability of antibiotic therapy and the relatively slow development of antibiotic discovery are becoming significant challenges in tackling this AMR. The discovery of antibiotics reached its peak in the 1950s and 1960s, the golden age of antibiotics. Unfortunately, only a small number of antibiotics have been introduced into the market since the late of 1980s, indicating the quiescence era of antibiotic discovery [9]. Based on the WHO’s 2020 Annual Review, a total of 43 antimicrobial agents are in the clinical trial phase, while more than 150 antimicrobial agents are still in the preclinical stage [10]. Drug-repurposing, use of clinically approved drugs with different indications to create new indications, might address this antibiotics deficiency. For example, N-acetylcysteine, known as a mucolytic, yields antimicrobial activity against several pathogens when administered alone [11] or with colistin [12,13]. In addition, we also need to think about the impact of COVID-19 on the AMR situation. Antibiotic misuse in COVID-19 patients will ruin antibiotic stewardship and management of AMR in clinics, leading to more AMR cases [14,15]. Even worse, COVID-19 patients living in areas with a high prevalence of AMR have to deal with both of these problems [15]. Rapid identification of this co-infection and priority setting in antibiotic prescription will preserve the lives and availability of current antimicrobial therapy. Although all those strategies might be beneficial in facing the current AMR situation, without the development of new antibiotics, one day, the choice of drugs for AMR therapy will also run out. Thus, the discovery of new antimicrobial compounds from various sources is an essential strategy to counteract AMR. This review summarizes the discovery of novel and prospective antimicrobial candidates, and also discusses pharmaceutical drug delivery approaches that can be used to prevent and/or to mitigate AMR. The latter is essential to prevent further development of AMR and at the same time can support the rational use of antimicrobials.

## 2. Antimicrobial Resistance: Current Problems and Its Mechanism

The emergence of antimicrobial resistance has been seen as one of fatal yet unresolved major medical problems, causing a heavy blow to healthcare systems worldwide. Factors driving the emergence of AMR are: (1) antibiotic misuse due to lack of compliance in patients or prescription error, (2) overuse of antibiotics due to overprescribing by physicians and high demands from patients, and (3) extensive agricultural use either as a food additive, prevention, or therapy, which leads to inappropriate disposal into the environment [16,17,18]. In addition, traveling also has a significant role in disseminating AMR throughout the world through skin contact or insect or animal bites during travel [19,20,21]. The most obvious example of the spread of infectious diseases through travel can be seen in the era of the COVID-19 pandemic [22,23].

The Interagency Coordinating Group on Antimicrobial Resistance implied in their 2019 report that around 10 million deaths each year will occur by 2050 if we cannot handle this AMR situation [2]. In addition to endangering lives, AMR can also impact the economy—especially in the health sector, due to the increased length of hospital stay, elevated treatment costs, failure of therapy, and the development and production of secondary therapy line drugs [24]. CDC estimates that about 55 billion is lost due to AMR in hospitals and lost productivity [8]. As a result, it is estimated that there poverty will be forced on about 24 million people by 2030 [2].

Like other organisms, bacteria have certain mechanistic systems for survival. The occurrence of resistance to antimicrobials itself is an evolutionary event [25]. However, inappropriate use of antibiotics worsens the condition, making antimicrobial resistance difficult to control [26]. The emergence of resistance in bacteria can occur from within the bacterial species itself, which is known as intrinsic resistance. Intrinsic resistance occurs naturally and is independent of previous exposure to antimicrobial compounds. Resistance can also occur due to externally acquired causes, called extrinsic resistance. Under certain conditions, bacterial cells can undergo horizontal gene transfer from the environment. This transfer of resistant genes can occur permanently or temporarily [27,28,29].

There are four main mechanisms for the occurrence of antimicrobial resistance in bacterial cells, including: (1) drug enzymatic inactivation; (2) drug uptake inhibition; (3) drug target modification; and (4) drug removal by active efflux. Intrinsic resistance uses uptake inhibition, inactivation, and efflux mechanisms. Meanwhile, extrinsic resistance can occur in the form of target modification, inactivation, and efflux [28,29,30].

Enzymatic inactivation of antimicrobial compounds can be achieved in two ways, including drug degradation or chemical group transfer. Enzymes that play a role in antimicrobial modification are divided into three main groups based on the type of reaction, namely hydrolases, transferases, and redox enzymes [31]. The β-lactamases are the best-known example of hydrolase enzymes. The substrates include the four classes of antibiotics that have a β-lactam ring, namely penicillin, cephalosporins, carbapenems, and monobactams [31]. These classes of antibiotics are also the most widely prescribed, so the rate of resistance is also increasing rapidly. In general, β-lactamase works by destroying the β-lactam ring, a vital structure required in the inhibition of bacterial cell wall synthesis. β-lactamases are classified into four classes: A, B, C, and D. Class A, C, and D are serine β-lactamases, while class B belongs to the metallo-β-lactamase group [32,33]. In addition to the β-lactam group, degradation of antibiotics due to enzymes also occurs in the tetracycline group. The term *tetX* refers to the gene encoding a tetracycline-degrading enzyme which was first identified in *E. coli.* This enzyme is a type of NADPH-dependent oxidoreductase [34].

The occurrence of bacterial resistance to antibiotics can also be achieved through the inhibition of drug uptake into bacterial cells. Gram-negative bacteria have a thicker lipopolysaccharide layer than their Gram-positive counterparts. This makes Gram-negative bacteria develop resistance more easily, especially to hydrophilic compounds [35]. However, this does not mean that drug uptake-related resistance is not found in Gram-positive bacteria. Vancomycin-resistant *S. aureus* exhibits thickening of the cell wall by an unknown mechanism, thus inhibiting drug entry [36]. Another interesting mechanism responsible for drug uptake inhibition is through biofilm formation. Biofilms are colonies of bacterial cells amassed in one place, forming an extracellular matrix (EMC). These EMCs are composed of a variety of bacterial secretions, including proteins, polysaccharides, and extracellular DNA [37,38]. The most common example is the biofilm of *P. aeruginosa* in the lung in cystic fibrosis [39]. The formation of a biofilm will strengthen the bacterial defenses, slowing the penetration of antibiotics into bacterial cells [38].

There are various types of antibiotics based on their mechanism and target of action. Therefore, there are also various problems related to resistance when it comes to where the antibiotics act. Another type of resistance mechanism is via drug target modification. One example of such resistance mechanism has been documented in the cases of penicillin and other β-lactams, particularly in a form of structural change of the Penicillin-binding protein (PBP). PBP plays a role in the synthesis of peptidoglycan in Gram-positive bacterial cells. Changes in the structure and expression of this protein on the cell surface will affect the effectiveness of -lactam antibiotics [40,41]. Meanwhile, for antibiotics that target ribosomes, mutations and other changes in the ribosomal subunit will affect drug binding. For example, the *erm* gene codes for the enzyme responsible for the methylation of the ribosomal subunit, making bacteria resistant to aminoglycosides and macrolides [42,43]. Resistance can also occur to fluoroquinolones due to changes in the DNA-gyrase and topoisomerase IV enzymes that play a role in DNA synthesis [44]. Changes in enzymes that play a role in folate biosynthesis will also cause resistance to sulfonamide and trimethoprim groups [45].

Another thing to consider is the presence of efflux pumps as a form of bacterial defense against environmental stressors [46]. There are five classes of efflux pumps that are important in the development of bacterial resistance. Three of them are H^+^-dependent pumps, namely resistance-nodulation-cell division (RND), major facilitator superfamily (MFS), and small multidrug resistance (SMR) [46,47]. RND is only found in Gram-negative bacteria and is a multi-component pump. In addition, RND acts on many substrates, and can be active against multiple drugs [48]. MFS is involved in the mechanism of resistance to macrolides and tetracyclines [49]. SMR, which is hydrophobic and has few substrates, plays a role in the efflux of β-lactams and some aminoglycosides [50]. There is also a multidrug and toxic compound extrusion (MATE) family that uses Na^+^ as an energy source. MATE is involved in the mechanism of resistance to many fluoroquinolones and several aminoglycosides [51]. Lastly, the ATP-binding cassette transporter (ABC transporter) family is associated with resistance to tetracyclines and fluoroquinolones that can occur in *V. cholerae* [52].

## 3. Current State of Antimicrobial Drug Discovery

**Figure 1:**
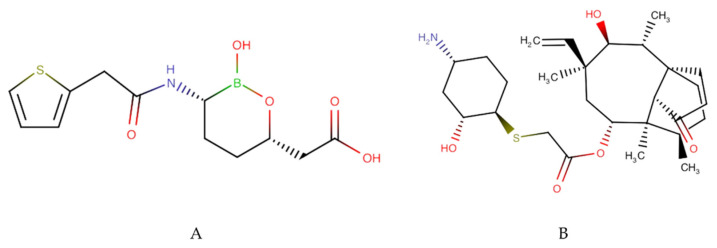
Chemical structures of (**A**) vaborbactam and (**B**) lefamulin.

The discovery of new antibiotic compounds and resistance modifying agents (RMA) has a high urgency in mitigating antimicrobial resistance. Research aimed at this is being carried out extensively all over the world. However, a long process is required before new compounds are given marketing authorization and can be commercialized. Based on data received by the WHO, there are 11 new antibiotics that have been approved for market in the period 2017–2020. Most of these compounds are derivatives of a class of antibiotics with a well-known mechanism of action, including the fluoroquinolones and tetracyclines [10]. Nevertheless, it is important to note that two of the compounds, vaborbactam and lefamulin (Figure 1), are listed as new types of antibiotics.

Vaborbactam, a beta-lactamase inhibitor, has been used clinically for the treatment of complicated urinary tract infections (cUTI) in combination with meropenem. Vaborbactam has good inhibitory activity against serine beta-lactamases, including *Klebsiella pneumoniae* carbapenemase (KPC). Currently, therapy using meropenem-vaborbactam is only given parenterally at a 1:1 ratio. Reported clinical data so far indicate this drug combination is well tolerated and effective against resistant bacteria [53]. Meanwhile, another drug known as lefamulin, is a new class of pleuromutilin antibiotics. Its mechanism of action is achieved through the alteration of protein synthesis on the ribosomes of bacterial cells. Clinical data show comparable effectiveness with fluoroquinolones in the treatment of community acquired pneumonia (CAP) [54,55]. The complete list of the eleven drugs that have been commercialized and have received U.S Food and Drug Administration (US FDA) and European Medicines Agency (EMA) approval since 2017 can be seen in Table 1.

**Figure 2:**
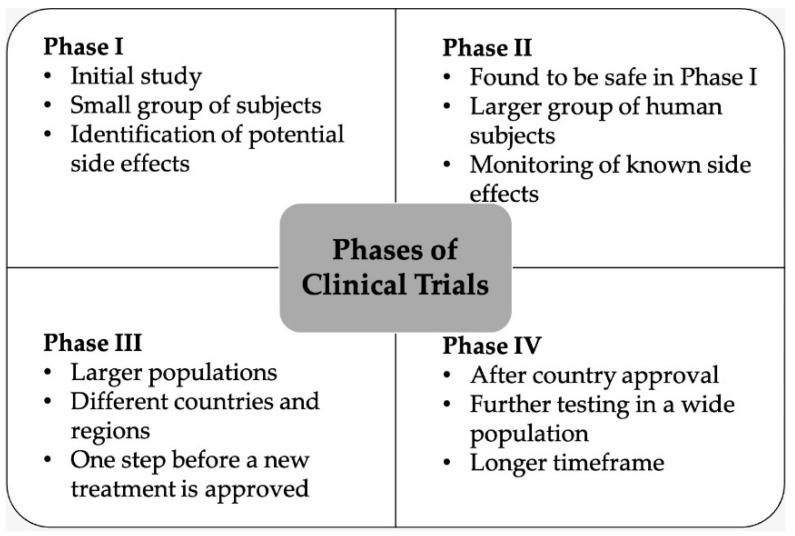
A summary of the phases of clinical trial according to the WHO.

Due to the prediction that problems related to AMR will continue to increase from year to year, the search for new antimicrobials continues. Scientists from all parts of the world are working hard on the discovery of compounds to overcome AMR. The WHO has listed several types of microorganisms as priority targets and special attention has been given to them. In the latest WHO report on antibacterial agents in clinical and preclinical development, the discovery of new antibiotic compounds under clinical testing is categorized into two groups, namely traditional antibiotics and non-traditional antibiotics. The clinical trial is a crucial stage to ensure the efficacy and safety of new drug compounds before they are marketed. Clinical studies on a substance consists of four phases, of which three phases must be completed before the agent can be commercialized. A summary of the phases of clinical trial according to the WHO can be seen in Figure 2.

The term traditional antibiotics refers to classes of antibiotics that are commonly used and whose mechanism of action is well known. There are 26 new traditional antibiotic compounds under clinical development that are reported to have activity against WHO priority pathogens. The WHO priority pathogen list is further divided into three categories, namely critical, high, and medium (Table 2). WHO has also set four criteria for innovation in new drug discovery, including: (1) no cross-resistance with other antibiotics, (2) new chemical class(es), (3) new target(s), and (4) new mechanism of action(s). Of the 26 compounds, only seven of them fulfill at least one of these criteria. Below, we will discuss antibiotics currently in clinical testing by their classes.

### 3.1. Beta-Lactams

Beta-lactams, or β-lactams, are the largest and most widely prescribed class of antibiotics, consisting of penicillin, cephalosporin, carbapenem, and monobactam derivatives [71]. As previously explained, the mechanism of resistance to this class of drugs is the production of the enzyme beta-lactamase by microorganisms. This enzyme can hydrolyze the beta-lactam ring, rendering the drug inactive [32,71]. One strategy to overcome AMR in beta-lactam drugs is a combination with beta-lactamase inhibitor (BLI) compounds. Currently, several drugs belonging to the beta-lactam/beta-lactamase inhibitor class are under clinical testing [32,72].

Two of them are single beta-lactams, namely sulopenem and benapenem. Sulopenem, a thiopenem derivative, is a drug that has been described since 1989, but development was discontinued in the 1990s. Due to the urgent need for drugs capable of inhibiting Gram-negative bacteria, studies regarding these drugs have been reopened. Sulopenem is currently in phase 3 clinical trials for its intravenous preparation and oral prodrug form, sulopenem-etzadroxil. This drug is reported to have good activity against extended-spectrum beta-lactamases (ESBL)-producing bacteria [73,74]. Benapenem is a carbapenem derivative that has only been clinically developed in China. This drug has now completed phase 2 clinical trials. Cross-resistance with other carbapenem drugs is reported [75].

**Figure 3:**
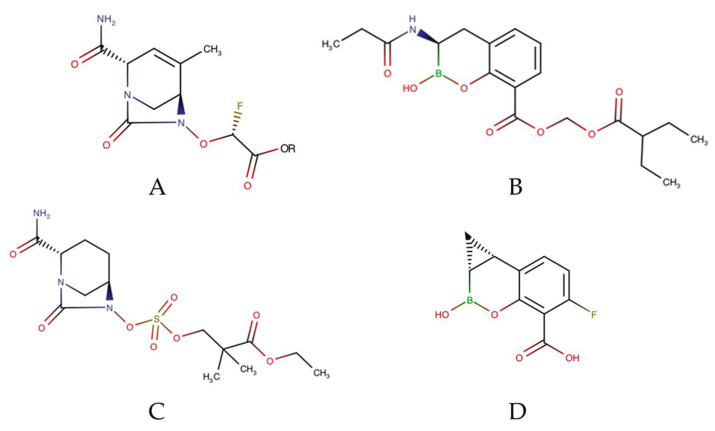
Chemical structures of (**A**) ETX0282, (**B**) VNRX-7145, (**C**) ARX-1796, and (**D**) QPX7728.

Beta-lactamase inhibitors (BLIs), currently in clinical trials, are divided into several classes, including boron-based BLI; diazabicyclooctanes; and penicillin-based sulfones [76]. Most of these compounds are only active against serine β-lactamases (class A, C, and D), but only a few have activity against metallo β-lactamases (class B). Almost all diazabicylooctanes-derived BLIs have intrinsic antimicrobial activity due to their ability to inhibit PBP2 [72]. A sulbactam–durloactam combination is currently in phase 3 clinical trials for infections caused by carbapenem-resistant *Acinetobacter baumannii* (CRAB). Sulbactam is known to have intrinsic activity against *A. baumannii*. However, due to the variety of beta-lactamase enzymes produced by this resistant species, its antibacterial potential is limited. Durloactam is a BLI with a broad spectrum of beta-lactamase classes A, C, and D, so it can support the activity of sulbactam. In vitro studies showed much lower resistance of *A. baumannii* to this combination when compared to amikacin, ampicillin–sulbactam, meropenem, and cefoperazone–sulbactam [77,78]. Three BLIs are being clinically developed in combination with cefepime, namely taniborbactam and enmetazobactam (phase 3), and zidebactam (phase 1). Cefepime-taniborbactam and cefepime-enmetazobactam were studied for the treatment of cUTI. Meanwhile, zidebactam has activity against *A. baumannii*, *P. aeruginosa*, and several *Enterobacterales*. Other BLIs include nacubactam (with meropenem), ETX0282 (with cefpodoxime), VNRX-7145 (with ceftibuten), ARX-1796, and QPX7728 (with an undisclosed β-lactam QPX2014) (Figure 3).

### 3.2. Tetracyclines

Tetracyclines are broad-spectrum antibiotics that work by inhibiting bacterial protein synthesis. The mechanism of resistance to tetracycline can be achieved through three main mechanisms, namely ribosomal protection, drug efflux, and enzymatic inactivation [79]. The discovery of synthetic and semisynthetic tetracycline derivatives by chemical modification in the last few decades demonstrates the potential of this class to be explored as a treatment for AMR. Tigecycline, approved for clinical use since 2005, is a parenteral semisynthetic glycycline that has been reported to have good activity against several resistant microorganisms. Two other tetracycline derivatives have also recently received approval, namely eravacycline and omadacycline for oral and intravenous use. Meanwhile, three tetracycline derivatives were recorded in phase 1 of clinical trials, namely KBP-7072, TP-271, and TP-6076. TP-271 and TP-6076 are synthesized by Tetraphase Pharmaceuticals. TP-271 is reported to be susceptible to *tet* (*A*) and *tet* (*X*), tetracycline-inactivating enzymes. TP-6076 has a slightly different chemical structure from other tetracycline derivatives and has activity against carbapenem-resistant Enterobacteriaceae (CRE) and CRAB [80].

### 3.3. Aminoglycosides

**Figure 4:**
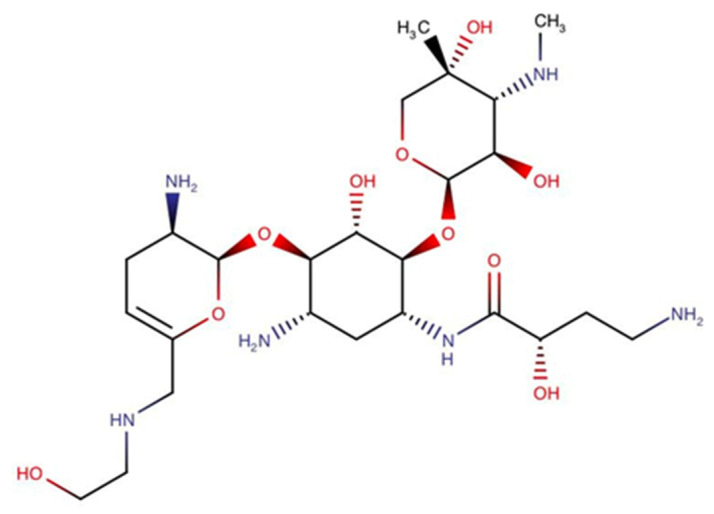
Chemical structure of plazomicin.

Just like tetracycline, aminoglycoside antibiotics also work by inhibiting protein synthesis in bacterial cells. However, over time, resistance also occurs to this class of antibiotics. Resistance to aminoglycosides is caused by the production of aminoglycoside-modifying enzymes (AMEs) and bacterial ribosome-modifying enzymes (16S rRNA methylase). Plazomicin (Figure 4), a newly approved drug, is stable in the presence of many types of AME, but is susceptible to 16S rRNA methylase [81]. In addition, the classic problems associated with the use of aminoglycosides, such as nephrotoxicity and ototoxicity, are still encountered with these drugs [79]. EBL-10031 (apramycin), an aminoglycoside previously used only for veterinary purposes, is currently in phase 1 clinical trials. Due to its unique chemical structure, it is resistant to a variety of clinically relevant AMEs, even to RNA methyltransferase, reducing its resistance [82]. In addition, this drug is also reported to have lower toxicity than other aminoglycosides [83].

### 3.4. Macrolides

Macrolides have bacteriostatic activity through inhibition of protein synthesis at the 50S ribosomal subunit. The most common mechanism for the occurrence of resistance in macrolides is RNA methylation reaction that changes the structure of the drug binding site on the ribosome [84]. A fourth-generation macrolide, solithromycin, has good activity against resistant microbes, especially when compared to the previous generation macrolides. Two phase III clinical trials for CAP and one for gonorrhea have been completed for this drug [85,86]. However, this drug did not get approval from the US FDA because of its worrisome side effects on the liver [87]. Nafithromycin, a ketolide derivative, has shown good susceptibility to CABP-causing bacteria, and is now entering phase 3 clinical trials [88].

### 3.5. Topoisomerase Inhibitors

Fluoroquinolones are part of this class of antibiotics that are widely used. However, two new topoisomerase II inhibitor compounds have been developed which have distinct structures to that of fluoroquinolones. Additionally, they act on different drug binding sites so that resistance-related issues can be overcome. Zoliflodacin is currently in a phase 3 clinical trial for uncomplicated gonorrhea [89]. Meanwhile, gepotidacin is being developed for uncomplicated UTI and uncomplicated gonorrhea [90].

### 3.6. Other Newly Developed Antimicrobial Classes

The development of antimicrobial compounds with new mechanisms of action is considered important in overcoming AMR. Some of these classes include bacterial enoyl-ACP reductase (FabI) inhibitors and the filamenting temperature-sensitive mutant Z (FtsZ) inhibitors. FabI is an enzyme that plays an important role in the final stages of fatty acid biosynthesis in bacteria cells. Afabicin, administered intravenously (i.v.) and orally, is a FabI inhibitor currently under phase 2 clinical trials for acute bacterial skin and skin structure infections (ABSSSI) and bone and joint infections. This drug acts specifically on Staphylococci and has no activity against other microorganisms [91]. Meanwhile, FtsZ inhibitors offer a new mechanism of action especially in overcoming AMR because of their ability to target FtsZ proteins that are vital in bacterial cell division. A prodrug of TXA-707, TXA-709, is currently in clinical trials. This drug has good activity against methicillin-resistant *S. aureus* (MRSA) [92].

## 4. Newly Prospective Pharmaceutical Candidates to Mitigate Antimicrobial Resistance

The following section will discuss the development of several novel AMR agents and the demonstration of their effectiveness, at least in vitro. This section will be divided into two topics: natural antimicrobial agents and synthetic antimicrobial agents.

### 4.1. Antimicrobial Candidates Sourced from Natural Products

The history of antibiotic discovery began from nature. In fact, the first antibiotic agent was discovered from a living organism. After that, the golden age of antibiotic discovery lasted 20 years, with most of the compounds being derived from natural resources [16]. Even now, natural antimicrobials still attract attention, either as an antibiotic candidates or template molecules for synthetic antimicrobials. This section will discuss the development of natural antimicrobial agents over the last few years.

#### 4.1.1. Antimicrobial Peptides (AMPs)

Antimicrobial peptides, small proteins with diverse biological activities, are considered one of the most dominant antimicrobial candidates in this current pipeline. Around 4500 sequences of AMPs are stored in the Data Repository of Antimicrobial Peptides (DRAMP) [93]. Although most antimicrobial peptides are currently obtained through synthesis, antimicrobial peptides can also be isolated from microorganisms, animals, and plants [94]. Bacteriocin—ribosomal AMPs synthesized by bacteria—is one of the most well-known AMPs and is widely used as a food preservative for a long time [95]. A recent discussion by Soltani et al. (2021) presented the potential of bacteriocin not only in food applications but also in the medical field; however, the safety and toxicity of bacteriocin became the highlight of the discussion [96]. Insufficient data regarding the safety and toxicity of bacteriocin will limit its broad use in industrial settings; therefore, further research about these matters is suggested [96]. Another example of natural AMPs is TPDSEAL, plant AMPs isolated from *Porphyra yezoensis* seaweed, showing bactericidal activity against *S. aureus* [97]. In addition to more AMPs sources, insects can be utilized to source novel AMPs with antibacterial and antifungal effects [98]. Lubelicin isolated from bovine rumen could kill MRSA strains after 30 min of exposure and had low cytotoxicity [99]. Several problems like stability, degradation by oxidation or proteolytic enzymes, relatively low half-life, toxicity, and hemolytic activity compared to antibiotics become significant obstacles in translating natural-derived AMPs to industrial settings [95,100]. However, the utilization of bioengineering techniques to produce more stable AMP-like compounds (e.g., peptidomimetics, which will be discussed later in this review) might offer an alternative solution to this problem.

#### 4.1.2. Antimicrobial Agents Sourced from Microorganism

Since the finding of the first antibiotics by Fleming in 1928, microorganisms have become one of the most exploited sources in the discovery of new antimicrobial compounds. Taking one example of natural antibiotics, polymyxin E, also known as colistin, is still favorable as an antimicrobial therapy for drug-resistant microorganisms in the clinic [101]. Though there are plenty of resources being funneled into finding new antimicrobial agents, filamentous saprophytic microbes living in diverse environments, famously known as Actinomycetes, are still popular, even nowadays. An example of recent findings of a novel antimicrobial compound from Actinomycetes is RSP 01, a lactone-based compound isolated from *Streptomyces* sp. RAB12 demonstrates a minimum inhibitory concentration (MIC) value ten times more potent than actinomycin-D against *C. albicans*, in addition to its antibacterial activity [102].

Although the main focus of antibiotic discovery in this current pipeline is still of non-extreme habitats of rare Actinomycetes, some researchers believe extreme conditions will be favorable to find more diversity in Actinomycetes and its unique metabolites. Several antimicrobial agents and other biologically active compounds sought from Actinomycetes have been discussed in many papers over the recent decades [103,104,105,106,107]. Those findings still consider Actinomycetes to be one of the largest producers of antimicrobial compounds. Currently, there is a shift of interest to rare Actinomycetes, especially the ones sought from various extreme environments like marine [108,109], thermophilic areas [110], desert [111,112], extreme cold habitats [113], and other harsh conditions [114] to expand the diversity of the unique metabolites obtained. Taking one example, Anthracimycin—a novel compound sought from marine Actinomycetes—showed potent bactericidal action against MRSA and *B. anthracis* as well as protective effects in animal models infected with MRSA [115,116]. Actinomycetes may remain a source of novel antimicrobial compounds in the coming years.

#### 4.1.3. Antimicrobial Agents Sourced from Plants

In recent years, searching for new antimicrobial compounds from medicinal plants has remained a trend among researchers due to the diversity of secondary metabolites. Medicinal plants can be utilized as herbs, extracts, or plant-derived compounds. For example, a research report on plant extracts of *Moringa oleifera* and *Matricaria recutita* implied their potential to prevent the growth of clinical isolates of MDR, XDR, and PDR-bacteria with MIC value range of around 7.8–62.5 mg/mL [117]. Another plant-based compound that is studied the most is curcumin. This phenolic compound exhibits a broad biological spectrum and various pharmacological effects such as anti-infective agent. In fact, curcumin exhibits activity against numerous bacteria, fungi, and viruses [118]. Recent reports on the activity of curcumin have shown that this compound displays a selective activity; its sensitivity was observed to be greater against Gram-positive than Gram-negative bacteria [119]. The same study reported the activity of curcumin against AMR bacteria and fungi; even though the results was not so promising [119]. Synergistic interaction of extract or plant-based compounds and antibiotics to repurpose resistant antimicrobial drugs has also become a huge trend; several compounds were reported to have synergistic action when combined with antibiotics [120,121,122,123]. As discussed by Cheesman et al. (2017), this approach might be an excellent strategy to tackle AMR, mainly since the product development and testing of plant-based agents (specifically plant extracts) does not require a large amount of money, meaning that its delivery to the market can be accelerated [124].

#### 4.1.4. Bacteriophages

Bacteriophages (BPs) are DNA/RNA viruses with capsid-enveloped structures capable of infecting and killing bacteria [125]. Bacteriophages can be used as carriers in the treatment of AMR because of their ability to kill bacteria precisely without killing commensal bacteria in the body, as well as carriers for agents that can target the regulation of genes that play a role in the growth and/or survival of the target bacteria [126]. In addition, BPs are inexpensive, have higher safety and tolerability, and are easy to administer with effects limited to the infected area [125,127]. The efficacy of BPs as antimicrobial agents has been demonstrated in various studies. Alemayehu et al. (2012) reported that two BPs (φMR299-2 and φNH-4) isolated from wastewater treatment were able to kill clinical isolates of *P. aeruginosa*, including in its biofilm form in lung cell lines [128]. Drilling et al. (2014) reported similar results on the use of BPs in reducing biofilms of clinical isolates of *S. aureus* in rhinosinusitis patients [129]. In addition to single use, the combination of BPs and antibiotics has also been reported to show increased activity in several studies [128,130,131] where the efficacy is highly dependent on the type of antibiotic given, the administration order, and the target bacteria. The relationship between effectiveness and BPs is one of the exciting things to note, as exemplified in the study conducted by Dickey and Perrot [130], where the use of BPs before antibiotics affects the pathogen but is otherwise ineffective.

Challenges in developing BPs as antimicrobial therapy are also covered in various studies, ranging from the host range to the clearance of BPs by the immune system. Although it is one of the advantages of BPs, the specificity of action of BPs also limits their use in clinical practice [125]; the type of bacteria that infects the host must be known before administering BPs [126]. These challenges can be overcome by providing cocktails containing various BPs or using phage engineering to expand the host range of BPs [126]. Phage engineering using clustered regularly interspaced short palindromic repeats (CRISPR)/CRISPR-associated protein (Cas) can also maximize efficacy by targeting the pathogen’s resistant genes or virulent genes and reducing the immune response against bacteriophages [126]. Another problem is the contribution of bacteriophages in spreading antimicrobial resistance genes (ARGs), which has been proven by the discovery of resistant genes in BPs in several studies [125,132,133]. However, the prevalence of ARG transduction is relatively low, and there are indications of an overestimation of ARG abundance [125]. Another issue of BP utilization is the possibility of developing resistance to BP target bacteria; however, replacing BPs with other types can be a solution to this problem [134].

### 4.2. Synthetic Antimicrobial Compounds

Chemical synthesis plays an essential role in the early stages of antibiotic development; sulfonamides are one of the most widely used examples of synthetic antibacterials in clinical practice. Over time, the synthesis of antimicrobial agents has progressed. Chemical synthesis is not only used to produce antimicrobial agents from scratch (fully synthetic antimicrobial agents) but also to improve the chemical characteristics of antimicrobial agents obtained from natural materials (semi-synthetic antimicrobial agents) [135]. Synthetic biology using genetic engineering techniques gives enormous benefits in developing novel antimicrobial compounds, resulting in the rapid growth of this research field [95]. This section will discuss the development of synthetic antimicrobial agents over the last few years.

#### 4.2.1. Chemically Synthetic Antimicrobial Compounds

Several novel potential fully synthetic antimicrobial compounds have been reported over recent decades. Salina et al. (2014) synthesized novel 2-thiopyridines compounds, which were effective against both active and in vitro dormant models of *M. tuberculosis* (MTB) cells [136]. The results showed that all compounds were active against the tested cells—the most significant activity was observed in compound 11026115, which killed dormant MTB H37Rv in three different in vitro models with MIC 0.250 mc/mL [136]. Recently, Seethaler et al. (2019) reported their successful attempt to generate two novel thienocarbazole compounds with antimicrobial activity towards *S. aureus* and Enterococcus species [137]. The lowest MICs were 2 mcg/mL against *S. aureus* and 8 mg/mL against *Enterococcus* in vitro. Both compounds also demonstrated their effectiveness in reducing the survival rate of *S. aureus* and Enterococci species upon in vivo testing using the *Galleria mellonella* infection model [137].

Other chemical compounds, novel urea derivatives, were created using one-step amine reactions using thiocyanates in toluene [138]. The compounds were tested on *Enterococcus faecium*, *S. aureus*, *K. pneumoniae*, *A. baumannii*, *P. aeruginosa*, and Enterobacter species (ESKAPE bacteria) and two fungi (*C. albicans* and *C. neoformans*). Several compounds showed moderate to excellent activities against *A. baumannii*, *K. pneumonia*, *S. aureus*, and fungi *C. neoformans*, while moderate to poor activities were found in *E. coli*, *P. aeruginosa*, and *C. albicans*, indicating their prospective use as new antibiotic candidates, especially towards *A. baumannii* with the highest inhibition percentage of 94.5% [138]. The mechanisms of these compounds were yet to be determined.

Babii et al. (2018) successfully created CICI-flav—a novel tricyclic flavonoid compound—through a two-step-reaction using 5-bromo-2-hydroxphenacyl-N-N-diethyldithiocarbamate as the starting molecule [139]. This compound exhibits bactericidal activity against both Gram-positive and Gram-negative bacteria, as well as antibiofilm activity [139]. Its mechanism of action is related to the disruption of cell membrane integrity. A toxicity assay on Vero cells showed no or very low toxicity at effective concentrations [139].

Capracazamycins (CPZs) A–G are an example of incorporating semi-synthetic techniques to improve the physical characteristics of an antimicrobial agent. Caprazamycin B (CPZ-B) is a potent anti-tuberculosis drug. However, its poor solubility and the difficulty in separating the compound from a complex mixture become considerable obstacles in its development [140]. Takahashi et al. (2013) modified the caprazene (CPZEN) obtained from the CPZs fermentation media through acid treatment to produce novel CPZs compounds. These compounds reported good solubility in water and better activity against *M. tuberculosis* than the original CPZ-B [140].

#### 4.2.2. Peptidomimetics

Among all synthetic agents, major compounds studied are peptidomimetics—small molecules designed to mimic the biological activity or characteristics of a peptide. Peptidomimetics often possess improved bioavailability and metabolic stability while retaining activity and selectivity profiles resembling AMPs [93,141]. The most fundamental difference from peptidomimetics is the lack of an α-amino acid from its backbone structure [141]. One of the most studied peptidomimetics is ceragenins—a group of peptidomimetics derived from bile acid and cholic acid. Ceragenins has protease-resistant properties since it is not protein-based and selectively targets the bacterial cytoplasmic membrane. The main advantages of ceragenins are their activity against microorganisms resistant to high levels of antibiotics and their ease of preparation for large-scale production [142]. The most-reported first-generation ceragenin was CSA-13. Several studies demonstrated that this compound had no or low toxicity; therefore, it was deemed suitable as a new antibiotic candidate for humans [142]. In the study conducted by Bozkurt-Guzel et al. (2014), CSA-13 showed synergistic interaction against clinical isolates of CRAB with colistin, tobramycin, and ciprofloxacin with the highest synergistic effect shown when combined with colistin (55% of tested strain, *n* = 20) [143]. A comparative study about the antimicrobial activity of first-generation ceragenins and second-generation ceragenins (CSA-142 and CSA-192) showed CSA-13 was still more potent than the second-generation MIC90. However, second-generation ceragenins showed better results in the time-killing assay as they showed earlier time points than the first-generation ceragenins [144]. Even though CSA-13 shows better activity than CSA-142 and CSA-192, second-generation ceragenins might be more beneficial than the first-generation due to better stability, environmentally friendly, and easier preparation [144]. Another second-generation ceragenins—CSA-131—exhibited bactericidal and excellent antibiofilm properties against *P. aeruginosa* [145] and *S. maltophilia* [146] as well as a synergistic activity when combined with colistin against CRAB [147].

#### 4.2.3. Endolysins

Endolysins (lytic endopeptidases) are bacteriophage-derived hydrolases that caused the osmolysis of Gram-positive bacteria by degrading the peptidoglycan of bacterial cell walls [148,149]. During the lysogenic cycle, the virus will inject its genome into the bacterial genome as a prophage. Under stress conditions, this gene will enter the replication state to start the lysis cycle [149]. This compound was reported for the first time by Frederick Tworth in 1915; several endolysins have entered the clinical trial phase over the last decade [149,150,151,152]. Endolysin has become an attractive alternative for antimicrobial agents because its activity remains when administered exogenously. Most endolysins are active against Gram-positive bacteria and can cause rapid cell lysis and death because these bacteria lack outer membrane protection [148,149]. Endolysins targeting Gram-positive bacteria tend to have a narrow spectrum, while endolysins targeting Gram-negative bacteria tend to have a broad spectrum. The main advantage of endolysin is that it is a safer molecule because it does not interact with the host, unlike bacteriophages which may mutate or be annihilated by the host’s immune system [149]. Recombinant endolysin can be synthesized by cloning prophage DNA that encodes the production of endolysin in bacteria.

LysBC17, a recombinant endolysin prophage derived from the genome of *B. cereus* strain Bc17, was reported to have narrow-spectrum lytic activity against several *Bacillus* strains [153]. Swift et al. reported similar activity in PlyCP10 and PlyCP41 endolysins obtained from the genome of *Bacillus* bacteria [154]. LysPA26 and LysAB54—Gram-negative endolysins—demonstrate good antibacterial activity against Gram-negative superbugs such as MDR *P. aeruginosa*, *A. baumannii*, and *E. coli* [155,156]. Moreover, endolysin LysPA26 was reported to kill *P. aeruginosa* even in the form of a biofilm. Another endolysin, LysSAP26, exhibits broad-spectrum activity against resistant ESKAPE bacteria with MIC ranging from 5–80 mcg/mL [155]. Drug delivery becomes a drawback in the use of endolysin in therapy. As reviewed by Murray et al. (2021), endolysin modification using molecular biology techniques (e.g., chimeric lysin, artilysin, virion-associated lysin, etc.) combined with optimization of delivery systems may solve this problem in the future [149].

#### 4.2.4. Nanoparticles

Nanoparticles (NPs) are tiny, nano-sized particles with various biological, including antimicrobial, activities. Although their exact mechanisms are not exactly understood, NPs and NP-based materials still invite interest to be developed as antimicrobial agents [157]. Advantages such as simple preparation, excellent and varied antimicrobial activities, and its ability to be used as a carrier for antibiotic delivery are the reasons for the popularity of this material [157,158]. Nanoparticles, in particular, metal nanoparticles, can be prepared by using conventional chemical techniques or green synthesis techniques with the help of bacteria, fungi, and plants. The green synthesis of nanoparticles has attracted vast attention due to its ease of preparation, environmentally friendly nature, and cost-effectiveness [159]. Several antimicrobial actions of metal-based nanoparticles have been documented over the past few years, ranging from their ability to inhibit the growth of resistant bacteria, antibiofilm activity, inhibit quorum sensing activity, to act synergistically with antibiotics [157,158,160]. Among the metal nanoparticles developed, silver nanoparticles (AgNPs) are the most potent metal-based nanoparticles [160]. One example of AgNPs activity—antibacterial and quorum QQ activity against *K. pneumoniae*—was demonstrated by Indian researchers [161]. AgNPs also displayed antifungal activity against several fungi such as *C. albicans* [162], *Malassezia furfur* [163], and several fungal pathogens [164,165,166]. When combined with visible blue light therapy, the antimicrobial activity of AgNPs therapy increased at sub-mic concentrations and the best activity was shown when these agents were combined with antibiotics [167].

Although NPs and NP-based materials show promising characteristics and activities as antimicrobial candidates, several challenges need to be addressed upon introducing these agents in the clinic. Cytotoxicity occurs as a problem in therapy with AgNPs; local administration of NPs can overcome this problem [168]. Before utilizing NPs in treatment, host-NPs interaction, optimal dose, and administration routes must be assessed [160]. Another arising problem is the growing prevalence of bacterial resistance towards NPs, including *A. baumannii*, one of the most concerning AMR bacteria in the clinic [169]. New strategies are urgently required to preserve the efficacy of NP-based materials as antimicrobial agents, including proper use and disposal of such preparations and a better understanding of the mechanism of microbial resistance against NP-based pharmaceuticals.

## 5. Drug Delivery Approaches via Different Routes to Overcome Antimicrobial Resistance

The fast occurrence of antimicrobial resistance in pathogenic microorganisms has become an impending worldwide public health problem. Management with conventional antimicrobial agents frequently results in resistance increase because most of these antimicrobial agents work on intracellular targets. Moreover, the application of these agents does not keep their bacterial morphology intact. Consequently, they are extremely prone to advance resistance throughout mutation. Bacterial resistance problems have occurred in different parts of the body. Accordingly, several drug delivery systems have been discovered with various routes to deliver antimicrobial agents to overcome this resistance issue [170,171,172,173,174,175,176,177,178].

### 5.1. Oral Route

Oral administration has been known as the safest and most convenient route for any medication, especially for antimicrobials. The oral route is preferable for antibiotic treatment over the intravenous route due to several advantages, such as being cheap, easy to administer, and not requiring any healthcare professional intervention due to the absence of a needle during the treatment [179,180]. Oral antibiotics can be considered the first option to treat any non-emergency condition over other routes if the bioavailability is over 90% compared to the intravenous route [180]. Therefore, bioavailability becomes a significant concern for the scientist in designing and developing antibiotic formulations for oral administration. Poor bioavailability may result in resistance development when the antibiotics fail to achieve the adequate serum concentration required to kill the bacteria [181]. Low aqueous solubility and limited permeability are reported to become the main factors associated with poor bioavailability [181,182].

In order to enhance the low solubility of amoxicillin and levofloxacin, Ojha and Das developed a drug-loaded microbial biopolymeric nanocarrier. This system was developed using a microbial polyester, poly (3-hydroxybutyrat-co-3-hydroxyvalerate), to improve the curative antimicrobial bioavailability of both amoxicillin and levofloxacin. In this study, the formulations were prepared by utilizing a triple emulsion technique. The size of particles was found to be in a range of 5–100 nm, and a biocompatibility study showed that these nanoparticles are biocompatible and safe from cytotoxic effects. Based on the antibacterial activity against *E. coli* and *S. aureus*, an increment of nanoparticle concentration results in an increase in the inhibition zone as well as a significant decrease in the bacterial survival ratio [182].

**Figure 5:**
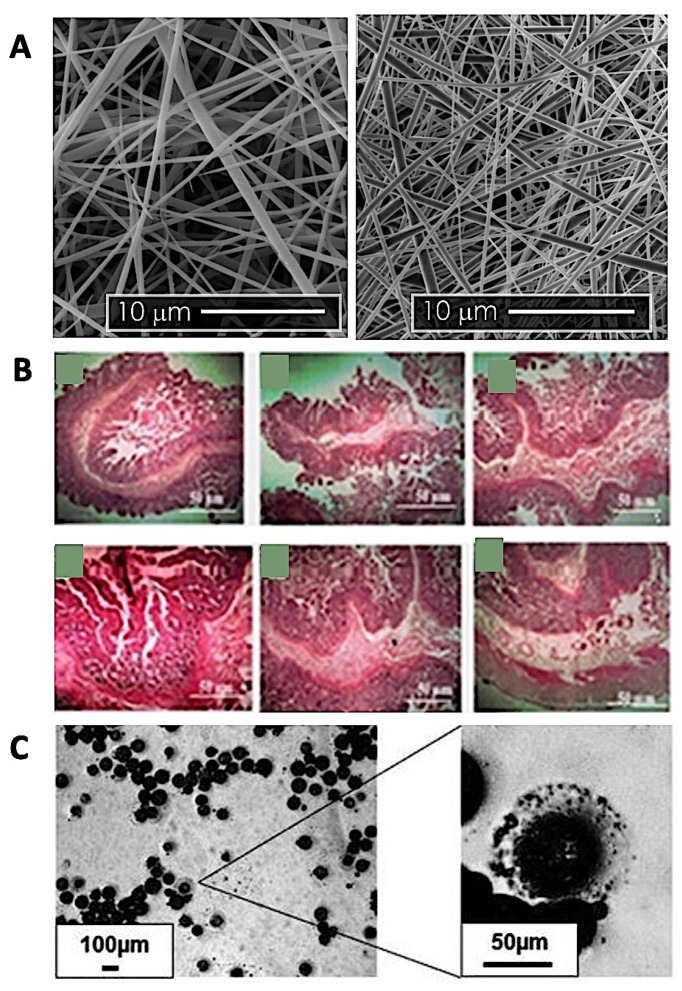
(**A**) Scanning electron microscope (SEM) images of cyclodextrin-antibiotic nanofibers [183]. Reprinted with permission from ref. [183]. Copyright 2020 Elsevier Inc. (**B**) Microscopic images of tissue histology after treatment with a self-emulsifying drug delivery system (SEDDS) in the intestine [184]. Reprinted with permission from ref. [184]. Copyright 2021 Elsevier Inc. (**C**) SEM images of encapsulated phage [185]. Reprinted with permission from Kerry and Danish (2021). Copyright 2021 Kerry and Danish.

An alternative way to increase the solubility of antibiotics is to form an inclusion complex with cyclodextrin. In a recent study, various antibiotics, such as kanamycin, chloramphenicol, gentamicin, and ampicillin, were individually formulated into nanofibers for oral delivery systems by utilizing the electrospinning technique [183]. The diameter of antibacterial nanofibers obtained was found to be in the range of 340 to 550 nm. All the solid-state characterization confirmed the inclusion complex formation between cyclodextrin and antibiotics (kanamycin, chloramphenicol, gentamicin, and ampicillin). Notably, the inclusion complexes of antibiotics in nanofiber particles demonstrated a rapid dissolution and release in water and artificial saliva. The antibacterial activity of cyclodextrin-antibiotic nanofiber was explored for 24 h using a zone-of-inhibition study performed on agar plates. The results showed that the inclusion complex nanofiber exhibited high bacterial activity against *E. coli*, whilst no inhibition zones were observed from each antibiotic’s control [183]. The scanning electron microscope (SEM) images of cyclodextrin-antibiotic nanofibers obtained by Topuz et al. (2021) is presented in Figure 5A.

In order to improve drug permeability, a self-emulsifying drug delivery system (SEDDS) has been documented by Arshad et al. (2021). This system has been investigated to enhance muco-penetration of ciprofloxacin. As a cell-penetrating peptide, poly-l-lysine was incorporated into the SEDDS of ciprofloxacin to enhance the system selectivity of the intracellular target of the *Salmonella enterica* serovar Typhi infection reservoir. This study showed that the SEDDS formulation was able to release 85% of ciprofloxacin in 72 h. An antimicrobial study showed that ciprofloxacin loaded in a SEDDS of poly-l-lysine, mannose, preactivated hyaluronic acid, and Pluronic F127 could minimize the survival rate of *S.* Typhi strains as well as show high killing activity compared to pure ciprofloxacin suspension. Moreover, the cellular uptake capability within the intracellular compartment of the macrophage has been confirmed by fluorescence imaging of the formulation. Therefore, this system could be a promising approach for eradicating *S.* Typhi intracellularly [184]. Microscopic images of tissue histology and results of treatment with SEDDS in the intestine can be seen in Figure 5B.

Nowadays, mitigation of antimicrobial resistance is not only focused on innovation of drug formulation. As a biological material, the enteric-targeted microbiome is difficult to formulate into an oral delivery formulation due to its high sensitivity to acid exposure. In order to overcome this issue, a study from Richards and Malik has successfully encapsulated an *E. coli* phage T3 in three different pH-responsive formulations for targeting the infectious bacterial cells. The encapsulated phages (Figure 5C) achieved a complete release within 30–45 min following exposure to different pH of simulated intestinal fluid (pH 5.5, pH 6, and pH 7). Moreover, in an acidic environment the encapsulate phages were stable, and the T3 phage can survive at low acidic pH. The stability study findings showed a 1 log decrease of phage viability in four weeks in refrigerator storage [185].

### 5.2. Parenteral Route

Parenteral administration of antimicrobial agents is recommended for patients who have severe and emergency conditions. Although the parenteral route is associated with needle-caused pain during administration, this route is still preferred due to manageable dosage, better bioavailability, and absence of first-pass metabolism [186]. Additionally, a previous study reported that the development of antibiotic resistance might be minimized by administrating the drug via parenteral injection [187]. Through this route, antibiotics can directly reach the systemic circulation and prevent the unnecessary exposure of antibiotics to the gut microbiota, resulting in a delay in antibiotic resistance [187].

In order to deliver an antibiotic through intravenous administration, Alcantara et al. developed nanostructured lipid carriers (NLCs) of mupirocin. This approach was able to produce particle in the size range of 99.8 to 235 nm. In this work, the NLCs exhibited the bacterial inhibition of *S. pyogenes* and *S. aureus* at 1.56 µg/mL and 0.78 µg/mL, respectively. These results were lower than free mupirocin, whose inhibition activity was 6.25 µg/mL and 1.56 µg/mL against *S. pyogenes* and *S. aureus*, respectively. An in vivo pharmacokinetic study in rabbits showed that plasma concentration and drug clearance were enhanced in NLCs formulations compared to free mupirocin. Therefore, this study provides a potentiality of mupirocin as a new parenteral antibiotic that was highly promising to be an alternative option for resistant bacterial infections [188].

A recent study has investigated the formation of inclusion complexes of doxycycline and HP-β-CD in order to develop a reconstitution formulation utilizing both freeze-drying and electrospinning techniques [186]. The results showed that the complex powder of doxycycline-HP-β-CD was fully dissolved in 1.5 mL water with the final concentration of 66.7 mg/mL. This concentration is seven times higher than similar marketed products. With regard to the manufacturing technique, electrospinning could produce more reconstitution powder in a day than freeze drying technology. Therefore, this approach could be a promising application in life-threatening conditions that require the rapid onset of doxycycline action [186].

Another problem pertaining to antimicrobial resistance is antibiotic overuse for intracellular infection treatment such as nontyphoidal *Salmonella* [189]. The inability of antimicrobial agents to access and enter the macrophages, where the pathogens reside, presents difficulties in overcoming this type of infection. Elnaggar et al. offer an alternative by developing nanotruffle formulations loaded with pexiganan and silver nanoparticles. This approach allows us to produce the particle with a specific size, 500–1000 nm, which is small enough for intravenous injection yet large enough to be selectively taken only by infected macrophages. In order to evaluate the antimicrobial activity of this approach, the nanotruffle formulations were evaluated using macrophages infected with different bacteria, namely *Shigella flexneri*, *S.* Typhimurium, *Listeria monocytogenes*, and MRSA. The results showed that the combination of silver and pexiganan in nanotruffle formulations against intracellular pathogens was significantly better than silver nanoparticles alone [190]. Through the rapid accumulation of nanotruffle formulations in the reticuloendothelial system following the intravenous administration, this study provides preliminary evidence and a high degree of promise that nanotruffle formulation has a viable application in the treatment of intracellular bacterial infection.

### 5.3. Inhalation Route

The inhalation route becomes a preferred route, especially for delivering several antibiotics targeted for any bacteria residing in the respiratory tract and organ. Indeed, pulmonary delivery enables us to deposit high concentrations in the lung, as a target site, while at the same time reducing systemic exposure [191]. However, the drug dose attained in the lungs can also be absorbed and reach systemic circulation via the pulmonary vasculature [192].

Devices are required to be incorporated with antibiotic formulations to facilitate drug delivery into the lungs. Generally, the devices are categorized into three: nebulizers, pressured metered-dose inhalers (PMDIs), and dry powder inhalers (DPIs). Firstly, nebulizers are developed for the drug in an aqueous solution or suspension form. This device is linked to a compressor as an external nebulization source for atomizing the solution or suspension into fine droplets. Secondly, PMDIs is combined with a propellant under pressure to generate an aerosol from suspension or solution. This device can produce a more uniform spray than is achieved with nebulizers [193]. Lastly, DPIs are developed for dispersing dry particles as an aerosol. The exact dose of drug administered using this device is controlled by the patient’s inspiratory airflow, duration, and inhaled volume [194]. To date, several approaches in dry inhaler powder have been proposed to be combined with DPIs for antibiotic administration in pulmonary-targeted therapy.

Tuberculosis is still a primary health concern, especially in developing countries. This disease can be cured by an antibiotic regimen that needs to be taken daily for up to six months [3]. However, failure in tuberculosis therapy and drug-resistant cases further complicates the treatment due to poor patient compliance for taking many pills daily. Consequently, several studies have been developed to improve tuberculosis treatment through pulmonary delivery to date. Chogale et al. reported that three main tuberculosis drugs, namely isoniazid, pyrazinamide, and rifampicin, were successfully formulated into dry powders incorporated with DPIs for direct pulmonary delivery. Initially, the three drugs were prepared into nanocrystals with a particle size in the range of 565 to 762 nm. The triple combination of dry powder inhaler was then developed by involving the nanocrystals and lactose into the formulations. The results confirmed that the dry powder formulation exhibited excellent flow properties and a fine particle fraction of 45%. In vivo lung deposition study showed that up to 80% of the doses were successfully administered to the lung, and approximately 20% of dose was retained over 24 h [195].

**Figure 6:**
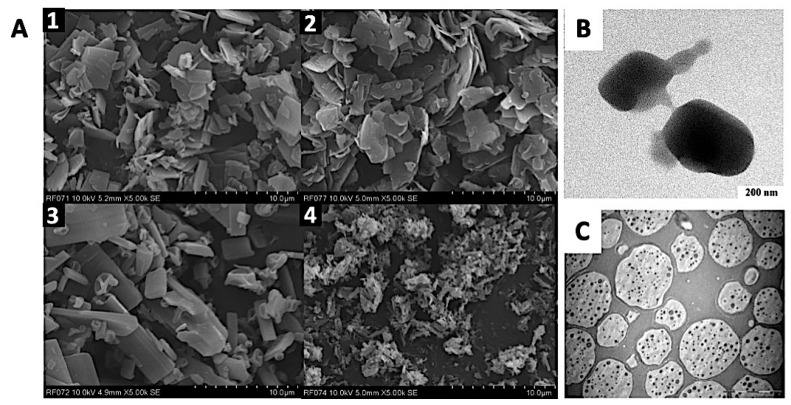
(**A**) Scanning electron microscope (SEM) images of rifampicin crystal from spray-dried samples using (1,2) ethanol, (3) methanol, and (4) water [196]. Reprinted with permission from ref. [196]. Copyright 2020 Elsevier Inc. (**B**) Transmission electron microscope (TEM) image of meropenem particles, scale bar: 200 nm [197]. Reprinted with permission from ref. [197]. Copyright 2020 Elsevier Inc. (**C**) SEM image of cross-sections of Poly(lactic-co-glycolic acid) (PLGA) microspheres containing levofloxacin [198]. Reprinted with permission from ref. [198]. Copyright 2018 Elsevier Inc.

Using other approaches for improved tuberculosis treatment, Berkenfeld et al. have recently demonstrated the potential of the spray drying technique to produce rifampicin in a stable dry powder formulation for direct pulmonary delivery. In this work, the authors investigated the stability of rifampicin spray dried from different solvents (ethanol, methanol, and water). The sample spray dried from ethanol was found to be stable in the storage condition over six months; on the contrary, samples from methanol and water exhibited a significant degradation and aggregation (Figure 6A) [196].

In order to combat another bacterial infection, meropenem (Figure 6B) was formulated into dry inhaler powder to achieve a better therapeutical effect due to stability issues in solution form as well as a short plasma half-life following intravenous administration [197]. The particle size was found to be in the range of 0.2–2 µm by utilizing a conventional micronization technique using mortar and pestle. The result of in vitro aerosol performance shows that the combination of L-leucine, lactose, and magnesium stearate could significantly improve the aerosol performance of meropenem up to 37.5%. Considering the desired therapeutic concentration in plasma is 25 µg/mL via injections, 200 mg of this dry powder inhalation formulation (containing 2.5% of meropenem) could be extrapolated to achieve a similar therapeutic result to an injection containing 500 mg meropenem [197]. Although this study offers a simple manufacturing process that is promising and industrially scalable, no antimicrobial or animal study supports the susceptibility and effectiveness of this approach against bacterial infection.

Poly(lactic-co-glycolic acid) (PLGA) microspheres containing levofloxacin for dry inhaler powder was developed by Gaspar et al. (Figure 6C). The microsphere formulations were evaluated for drug release and cytotoxicity. The selected microspheres formulation had controlled release properties and were well-tolerated by Calu-3 cells. Considering 60–70% of the levofloxacin is released from the PLGA-microsphere over 24 h, this means the formulation can be highly concentrated in the lung for a prolonged time. This approach could be beneficial for patients since the dosing frequency can be reduced and treatment efficiency can be improved [198].

### 5.4. Topical Route

Li et al. developed hydrogel formulations containing a combination of physically crosslinked antimicrobial agents possessing broad spectrum activities prepared from the complex reaction between two biodegradable polymers, namely PLLA-PEG-PLLA (PLLA = poly(l-lactide) and polycationic PDLA-CPC-PDLA (PDLA = poly(d-lactide, CPC = cationic polycarbonate) [199]. The synthesis process of PLLA-PEG-PLLA and PDLA-CPC-PDLA precursor triblock copolymers was carried out through organo-catalyzed ROP. As the macroinitiators, the authors used PEG and polycarbonate in the synthesis reactions. Via this reaction, the desired characteristic of polymer, namely well-controlled sequence sizes with the optimum degree of polymerization, was achieved. Afterwards, the precursors were reacted with trimethylamine through quaternization in order to attain the cationic polycarbonates. Importantly, due to the efficient combination of PLLA-PEG-PLLA and PDLA-CPC-PDLA precursor triblock copolymers, the authors were able to produce a succession of supramolecular hydrogel assemblies with lower critical solution temperature performance, shear-thinning performance, and abilities to disrupt biofilm at 37 °C [199]. In this study, the antimicrobial activity of the formulation was investigated against various types of pathogenic microorganisms, namely *S. aureus*, *E. coli*, and *C. albicans*. Additionally, several clinically isolated microorganisms were also used, namely methicillin-resistant *S. aureus*, vancomycin-resistant *enterococci*, *P. aeruginosa*, *A. baumannii* (resistant to most antibiotics), *K. pneumonia* (resistant to carbapenem), and *C. neoformans*. The results showed that the hydrogels were found to entirely eradicate growth and display complete killing effectiveness on all the microorganisms examined even though the cationic PDLA-CPC-PDLA polymers in solution exhibited poor antimicrobial performance [199]. Specifically, following the investigation of the mechanism of the antimicrobial activity, the disruption of membrane or cell wall of microorganisms was found to be the possible mechanism after the observation of morphology of microorganisms using scanning electron microscopy. Essentially, the optimum formulation of hydrogel developed in this study exhibited insignificant toxicity both in in vitro and in vivo evaluations.

Numerous polysaccharide derivates have been extensively applied as the main vehicles for the delivery of antimicrobial agents because of their biocompatibility and biodegradability [200]. For instance, Zumbuehl et al. developed hydrogels containing the broad-spectrum antifungal drug, amphotericin B, with dextran as the hydrogel vehicle. The dextran-based hydrogel laded with amphotericin B was able to kill *C. albicans* effectively within two hours. Importantly, the application of this formulation did not result in any hemolysis [201]. Using a similar approach, Glisoni el al. developed hydrogels containing thiosemicarbazones formulated from β-cyclodextrin for ocular delivery [202]. The antimicrobial activity evaluations showed that hydrogels laded with Thiosemicarbazone successfully eradicated *P. aeruginosa* and *S. aureus*, two bacterial strains that generally cause ocular infections [202].

In addition to the polysaccharide derivates, in the topical hydrogel formulation, poly(acrylate)s have also been applied as a vehicle. These polymers are well-known for their ability to specifically deliver antibiotics to the desired site, although these compounds have shown poor biodegradable characteristics. In this application, Jones et al. explored different types of hydrogels formulated via the copolymerization of methacrylic acid and N-isopropylacrylamide. In their study, various types of hydroxy methacrylates were used and chlorohexidine diacetate was used as the active ingredient [203]. The results showed that the optimum formulation was obtained by copolymerizing N-isopropylacrylamide with 2-hydroxyethyl methacrylate at a ratio of 1:1. The rapid and controlled release of chlorohexidine diacetate was attained specifically at 37 °C. Importantly, the formulation successfully inhibited the growth of *S. epidermis*.

Cheng et al. developed a combination of antimicrobial and antifouling agents incorporated into hydrogel formulations. This combination was established with the aims of avoiding the development of planktonic bacterial cells whilst maintaining clean surfaces [174]. In their study, salicylate as a mild antimicrobial compound was used as the active ingredient and formulated into a hydrogel prepared from the crosslinking reaction of a poly(carboxybetaine) (pCB)-containing methacrylate-base as its anion. The results showed that the hydrogel formulation was able to stop the proliferation of two bacteria strains, namely *S. epidermidis* and *E. coli* with 99.9% killing efficiency. Accordingly, this approach was found to have a potential application in wound dressings and surface coatings for biomedical apparatus. Remarkably, without the incorporation of salicylate, pCB-based hydrogel was only able to reduce the attachment of the bacteria without killing efficiency. In a follow-up examination, the authors continued using this approach and performed modifications of their study. They changed the use of the methacrylate-based chemically crosslinked hydrogel with a thermosensitive hydrogel. To achieve this purpose, they used thermosensitive polymer, poly(N-isopropylacrylamide) (PNIPAM) in the antimicrobial wound dressing formulation [204]. The results revealed that the addition of NIPAM into the formulation was able to produce a thermo-responsive hydrogel possessing in situ gelation abilities at body temperature. This could potentially result in suitable and desired properties for wound dressing purposes. In the microbial activity evaluation, the hydrogel formulation was found to completely eliminate the growth of *E. coli*. Importantly, the formulation did not show any cytotoxicity on mammalian fibroblast cell line COS-7 [204].

Yabanoglu et al. examined the antibacterial activities of various topical antimicrobial dressings, namely 1% silver sulfadiazine (SSD), 0.5% chlorhexidine acetate, 3% citric acid, and silver-coated dressing on a multi-drug resistant P. aeruginosa. The study was carried out in vivo in an infected full thickness burn wound model in rats. After a seven-day application period, 1% SSD and silver-coated dressing were found to be effective at reducing the viability of P. aeruginosa colonies in the infected rats. Therefore, these preparations could be considered effective agents in the treatment of burn wound infections. Importantly, in this study, there was no mortality found in any of the groups [170].

As an emerging approach, the formulation of antimicrobial agents into nanoparticles has been found to deliver higher amounts of these agents to the infected skin sites. Therefore, this could potentially overcome resistance possibility with low side effects. The incorporation of antimicrobial agents into nanoparticle formulations circumvent the microorganism resistance by avoiding the declined uptake and improved efflux of drug from the cells of the microorganisms, intracellular bacteria, and formation of biofilm [175].

**Figure 7:**
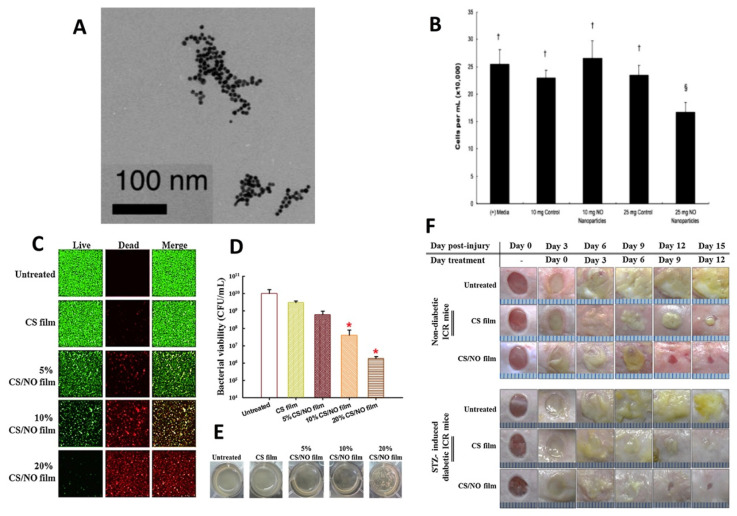
The morphology of NO-loaded nanoparticles observed using a transmission electron microscope (TEM) (**A**). Results of toxicity evaluations of nanoparticles containing NO, showing non-toxic activity in human fibroblasts († indicates that *p* > 0.05, § indicates that *p* < 0.0194) (**B**) [178]. Reprinted with permission from ref. [178]. Copyright 2008 Elsevier Inc. Anti-MRSA activity of film containing S-nitrosoglutathione (GSNO) observed using confocal microscope (**C**). The viability of MRSA following the application of GSNO film (**D**). The growth of MRSA in TSB medium in the presence of GSNO film (**E**). Bacterial viability (CFU measurement). Values are expressed as mean ± SD (*n* = 3); * *p* < 0.05 compared with the untreated group. (**E**) Macroscopic images of bacterial density in TSB medium. In vivo antibiofilm activity of GSNO film in non-diabetic and STZ-induced diabetic ICR mice (**F**) [206]. Reprinted with permission from ref. [206]. Copyright 2019 Elsevier Inc.

Friedman et al. formulated nitric oxide loaded nanoparticles for prospective application in antimicrobial treatment against MRSA and MSSA (Figure 7A) [178]. Following the determination of the MICs values, nanoparticles laden with nitrite oxide showed MIC values between 312 and 2500 μg/mL against MRSA and between 312 and 1250 μg/mL against methicillin-sensitive *S. aureus* (MSSA) strains. Importantly, the nanoparticles were found to be non-toxic in human fibroblasts (Figure 7B). In an in vivo study, an MRSA-infected wound was developed in murine model. The results showed that infected mice receiving nanoparticles containing nitric oxide exhibited drastically lower bacterial bioburden when compared to untreated mice or mice receiving nanoparticles. It has been hypothesized that nanoparticles laden with nitric oxide enhanced the wound healing properties by triggering minimum degradation of collagen. The results obtained here supported the notion that nanoparticles containing nitric oxide could potentially be utilized as an innovative topical antimicrobial dosage form for the effective management of skin and cutaneous infected wounds [205].

In another work related to nitric oxide, Choi et al. developed S-nitrosoglutathione (GSNO) into chitosan film for the treatment of bacterial biofilm infection caused by MRSA (Figure 7C–E). In this study, chitosan was selected due to its antimicrobial and anti-biofilm properties [206]. As an active ingredient, S-nitrosoglutathione has been recognized as an excellent nitric oxide (NO) donor to eliminate pathogenic biofilms and to improve wound healing behaviors. The results showed that the incorporated GSNO into chitosan films was able to control the release of NO for three days in simulated wound fluid. The film formulation containing GSNO-loaded films could result in more than three logs reduction in MRSA viability. Furthermore, this approach showed three-times greater anti-biofilm properties in comparison with the control formulation. In vivo study was carried out in MRSA biofilm-infected wounds in non-diabetic and STZ-induced diabetic mice. Importantly, in in vivo study, the film containing GSNO exhibited rapid biofilm removal and a decrease in wound size, collagen deposition, and rates of epithelialization when compared to the untreated and film without GSNO cohorts (Figure 7F) [206]. This study suggested that GSNO incorporated into films could be a favorable system for the management of infection associated with MRSA biofilms.

To date, microneedle delivery systems have been widely used to topically deliver antimicrobial agents into an infection area [176,207,208,209,210,211]. Permana et al. developed dissolving microneedles containing doxycycline nanoparticles. The nanoparticles were prepared using responsive polymers, namely poly(lactic-co-glycolic acid) and poly (Ɛ-caprolactone) coated with chitosan [173]. The incorporation of doxycycline into these polymers was able to specifically control the release of the drug in the presence of bacterial cultures. In an ex vivo biofilm model using MRSA and a resistant strain of *P. aeruginosa*, this approach was able to eradicate up to 99.99% of bacterial bioburden after 48 h of application. Similarly, the authors incorporated silver nanoparticles into bacterially sensitive microparticles prepared from poly (Ɛ-caprolactone). The microparticles were further formulated into dissolving microneedles. In comparison with dissolving microneedles containing silver nanoparticles without microparticle formulations and conventional cream formulations, around 100% of bioburdens of MRSA and resistance strain of *P. aeruginosa* were eliminated in an ex vivo biofilm model in rat skin after 60 h of the application of this system [207].

### 5.5. Transdermal Route

Transdermal delivery systems have been developed to deliver antibiotics to the systemic circulation in order to overcome the antimicrobial resistance issue. Rastoge et al. designed an innovative microemulsion formulation to deliver bacteriophage (T4), specifically lysing *E. coli* via the transdermal route [212]. This study was designed to overcome the resistance issue in treatment associated *E. coli*. In this study, the microemulsion formulation was done using pseudoternary phase diagrams. The compositions of the microemulsions were the combination of ethyl oleate, Tween 80: Span 20 and water as oil phase, and stabilizers as aqueous phase, respectively. The results showed that the microemulsion droplets were approximately 200 nm in size with a narrow size distribution. Importantly, the formulations possessed acceptable viscosity and surface tension. The transdermal ability evaluated by ex vivo permeation showed that up to 6.7 × 10^6^ PFU/mL of T4 were able to permeate across the skin from the microemulsion formulation. In vivo permeation study was performed in the *E. coli* challenged rats and 5.49 × 10^5^ PFU/mL of T4 was found in the blood of the rats. Furthermore, at the end of the study 2.48 × 10^5^ PFU/mL of T4 was found in germ free rats. Importantly, in the cohort where the rats infected with *E. coli* received microemulsion of T4, no mortality was found. On the other hand, when the infected rats did not receive the developed formulation, noteworthy mortality was found. Moreover, analyzed using histological and IL-6 immunofluorescence evaluation, the administration of microemulsion containing T4 exhibited a safety effect on the treatment. Therefore, this approach could potentially be utilized as an alternative treatment for the infections caused by antibiotic-resistant bacteria [213].

As a solution for infection in the neonatal, gentamicin was formulated into a microneedle approach delivered transdermally [214]. The dissolving microneedles were prepared using two water soluble polymers, namely sodium hyaluronate and poly(vinylpyrrolidone). The microneedles were found to have adequate mechanical and penetration properties. In vitro transdermal delivery showed that microneedles were able to deliver around 4.45 mg of gentamicin after 6 h. In an in vivo study using rats, it was found that dissolving microneedles could deliver gentamicin transdermally at therapeutic levels. Furthermore, Rodgers et al. evaluated the in vivo efficacy of gentamicin loaded dissolving microneedles in a murine model of *K. pneumoniae* bacterial infection [172]. The microneedles were applied to murine ears. The results showed that the bacterial bioburden in the nasal-associated lymphoid tissue and lungs in the infected mice administered gentamicin loaded microneedles decreased significantly in comparison with the untreated cohorts.

In order to increase the efficacy of vancomycin hydrochloride for MRSA treatment, Magdy et al. developed an ethosomes approach combined with iontophoresis to transdermally deliver vancomycin [215]. Ethosomes were prepared by a cold method using soy phospholipids, ethanol, and propylene glycol. Vancomycin loaded ethosomes exhibited excellent electrochemical stability. Furthermore, cathodal iontophoresis of negatively charged vesicles displayed the highest transdermal flux with a value of 550 µg/cm^2^/h in comparison with free drug solution. In this study, a Sprague Dawley rats infection model was prepared by producing mediastinitis using MRSA. The transdermal delivery developed in this study was compared to intramuscular administration and an untreated group. The in vivo efficacy study showed that the bacterial bioburden of the infected groups receiving intramuscular administration and transdermal administration were not significantly different and significantly higher compared to untreated group. Accordingly, the combination of ethosomes and iontophoresis were successfully able to deliver vancomycin transdermally [215].

Recently, with similar purpose, Ramadon et al. further developed two types of microneedles, namely dissolving and hydrogel-forming, to also deliver vancomycin transdermally [216]. All formulations were characterized, and it was found that the microneedles prepared possessed sufficient mechanical characteristics. Furthermore, in an ex vivo transdermal delivery study across the neonatal porcine skin, both types of microneedles successfully delivered vancomycin with the percentage of drug around 46.39 ± 8.04% and 7.99 ± 0.98% for dissolving and hydrogel-forming microneedles, respectively. In in vivo studies in a rat model, the administration of dissolving and hydrogel-forming microneedles resulted in the area under the plasma concentration time curve from time zero to infinity (AUC0–∞) values of 162.04 ± 61.84 and 61.01 ± 28.50 μg.h/mL, respectively. On the other hand, these values were significantly higher than that of the oral administration of vancomycin [216]. Therefore, transdermal delivery of vancomycin could be an alternative treatment for infectious diseases caused by MRSA.

### 5.6. Vaginal Delivery System

**Figure 8:**
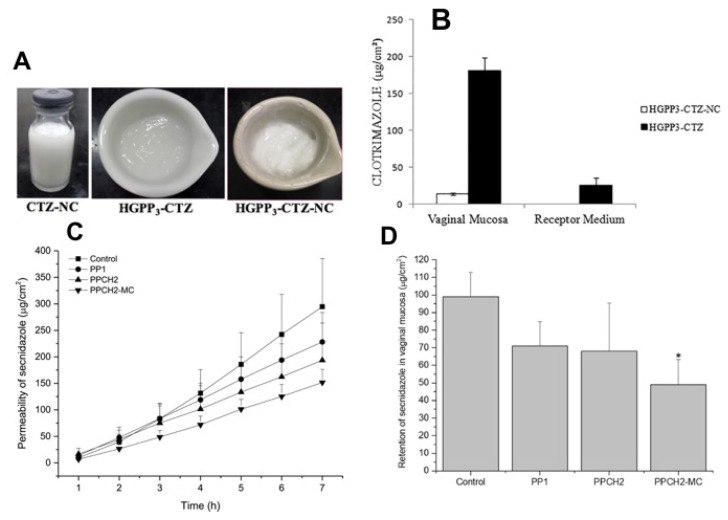
Hydrogel formulations containing clotrimazole nanocapsules (**A**). The results of ex vivo permeation and retention evaluations in vaginal mucosa of hydrogel formulations containing clotrimazole (**B**) [171]. Reprinted with permission from ref. [171]. Copyright 2017 Elsevier Inc. The results of ex vivo permeation (**C**) and retention evaluations in vaginal mucosa (**D**) of secnidazole loaded hydrogels [217]. * *p* < 0.05 compared with Control group. Reprinted with permission from ref. [217]. Copyright 2021 Elsevier Inc.

In order to assist in the delivery of clotrimazole for vaginal delivery, de Lima et al. developed nanocapsule formulations incorporated into hydrogel dosage forms (Figure 8A) [171]. In this study, Pemulen^®^ TR1 and Pullulan were used as mucoadhesive polymers. The mucoadhesive hydrogels obtained were found to have pH values close to vagina pH, indicating that the application of these gels would not cause any irritation to the vaginal mucosa. In spreadability evaluations, the clotrimazole-loaded nanocapsules in hydrogel formulations showed similar properties compared to free clotrimazole loaded hydrogel formulations. The mucoadhesive evaluations showed that pullulan with concentration of 3% showed an adequate mucoadhesive in cow vaginal mucosa. Importantly, this innovative approach could retain clotrimazole in the vaginal surface (Figure 8B). Accordingly, the hydrogel formulations containing clotrimazole nanocapsules and pullulan could potentially be used as an alternative treatment of vulvovaginal candidiasis.

In another study, Argenta et al. formulated thermosensitive-bioadhesive vaginal gel containing secnidazole [217]. This drug has been considered an alternative antimicrobial agent for infection caused by *Trichomonas vaginalis*. Additionally, this drug is also used to solve antimicrobial resistance issues of conventional therapy. In this study, the gel contained poloxamer 407 and poloxamer 188 as thermosensitive polymers and chitosan as mucoadhesive agents. The formulations showed desired mucoadhesive properties and sol–gel transition temperature with a rapid gelation time. Importantly, the thermosensitive-bioadhesive vaginal gel was able to reduce the permeability and increase the retention of secnidazole in comparison with control formulation (Figure 8C,D). With these results, this approach could be beneficial in the treatment of trichomoniasis.

Recently, to improve the solubility of itraconazole in vaginal delivery, this drug was formulated into solid dispersion loaded into gel flakes [177]. This approach was further incorporated into thermosensitive and bioadhesive vaginal gel. The formulation of itraconazole in solid dispersion was able to improve the solubility of the drug. Additionally, being formulated into gel flakes, the itraconazole was encapsulated with desirable entrapment efficiency and drug-loading capacity. The hydrogel formulation contained pluronic as a thermosensitive agent and HPMC as the mucoadhesive polymer. The results showed that the incorporation of solid dispersion-gel flakes of intraconazole into thermosensitive and bioadhesive hydrogel improved the localization of the drug in the vaginal tissue in an ex vivo study. Importantly, the formulation was found to have adequate mucoadhesive and thermosensitive properties. In an in vivo study using a vaginal candidiasis model of *C. albicans*-infected Wistar rats, this combination approach improved the antifungal activity of itraconazole in comparison with other treated groups [177].

## 6. Concluding Remarks and Future Perspectives

Escalation of infectious cases caused by antimicrobial resistant pathogenic microorganisms has become a significant threat worldwide. Currently available antimicrobial preparations, unfortunately, have not been properly utilized to address this issue. Given the rise of pathogen resistance and the inadequate supply of effective antimicrobials, we sought to discuss the importance of discovering novel synthetic or naturally occurring antibacterial compounds to manage antimicrobial resistance. Evidently, this issue is vital for humans’ wellbeing, thus innovative and meticulous actions on the application of various drug delivery approaches to deliver those novel antimicrobial compounds through numerous routes shall provide advantageous approaches to the management of infectious diseases. Nevertheless, there is a need to precisely design the delivery systems for different types of infectious diseases to prevent the emergence of antimicrobial-resistant pathogens towards the newly developed antimicrobial agents. In the end, careful considerations in the development of cost-effective novel antimicrobials and highly effective drug delivery systems shall offer substantial benefits for all parties, including research institutes, pharmaceutical industries, health providers, and public communities. Such cooperation will also provide a great scientific opportunity to connect basic findings (bench) to clinical implications (bedside) in the continuous effort to tackle the emergence of antimicrobial-resistant pathogenic microbes.

## Figures and Tables

**Table 1:** Newly developed antimicrobials since 2017.

Drug Name	Trade Name	Antibiotic Class	Administration Route	Indication(s)	Ref(s)
Cefiderocol	Fetroja (Shionogi)	Siderophore cephalosporin	iv	cUTI	[56]
Delafloxacin	Baxdela (Melinta)	Fluoroquinolone	iv; oral	ABSSSI; CAP	[57,58]
Eravacycline	Xerava (Tetraphase)	Tetracycline	iv	cIAI	[59]
Lascufloxacin	Lasvic (Kyorin Pharmaceutical)	Fluoroquinolone	iv; oral	CAP; otorhinolaryingological	[60,61]
Lefamulin	Xenleta (Nabriva)	Pleuromutilin	iv; oral	CAP	[62]
Levonadifloxacin Alalevonadi-floxacin	Emrok/Emrok O (Wockhardt)	Fluoroquinolone	iv; oral	ABSSSI	[63]
Omadacycline	Nuzyra (Paratek)	Tetracycline	iv; oral	CAP (iv); ABSSSI (iv;oral)	[64,65]
Plazomicin	Zemdri (Achaogen)	Aminoglycoside	iv	cUTI	[66]
Pretomanid	PA-824 (TB Alliance)	Nitroimidazole	oral	XDR- and MDR-TB	[67]
Relebactam + imipenem/cilastatin	Recarbrio (MSD)	BLI + carbapenem/degradation inhibitor	iv	cUTI; cIAI; HAP/VAP	[68,69]
Vaborbactam + meropenem	Vabomere (Melinta)	BLI + carbapenem	iv	cUTI	[70]

ABSSSI: acute bacterial skin and skin structure infections; CAP: community-associated pneumonia; cIAI: complicated intra-abdominal infection; cUTI: complicated urinary tract infection; HAP: hospital-associated pneumonia; iv: intravenous; MDR: multi-drug resistant; VAP: ventilator-associated pneumonia; XDR: extensively drug-resistant.

**Table 2:** The WHO priority pathogen list [10].

Critical Priority	High Priority	Medium Priority
Carbapenem-resistant *Acinetobacter baumannii* (CRAB)	Vancomycin-resistant *Enterococcus faecium*	Penicillin-non-susceptible *Streptococcus pneumoniae*
Carbapenem-resistant *Pseudomonas aeruginosa* (CRPA)	Clarithromycin-resistant *Helicobacter pylori*	Ampicillin-resistant *Haemophilus influenzae*
Carbapenem- and 3rd gen. cephalosporin-resistant Enterobacteriaceae	Fluoroquinolone-resistant *Salmonella species*	Fluoroquinolone-resistant *Shigella species*
	Vancomycin- and methicillin-resistant *Staphylococcus aureus*	
	Fluoroquinolone-resistant *Campylobacter species*	
	3rd gen. cephalosporin- and fluoroquinolone-resistant *Neisseria gonorrhoeae*	

## Data Availability

Available data are presented in the manuscript.
